# Supplementary material for: Development of an In Vivo Extended-Spectrum Cephalosporin-Resistant Escherichia coli Model in Post-Weaned Pigs and Its Use in Assessment of Dietary Interventions
Source: Animals (Basel). 2023 Mar 7;13(6):959. doi: 10.3390/ani13060959 (PMC10044249; doi:10.3390/ani13060959)
Supplement: Supplementary file 1 [file animals-13-00959-s001.zip › animals-2224967-supplementary.pdf]

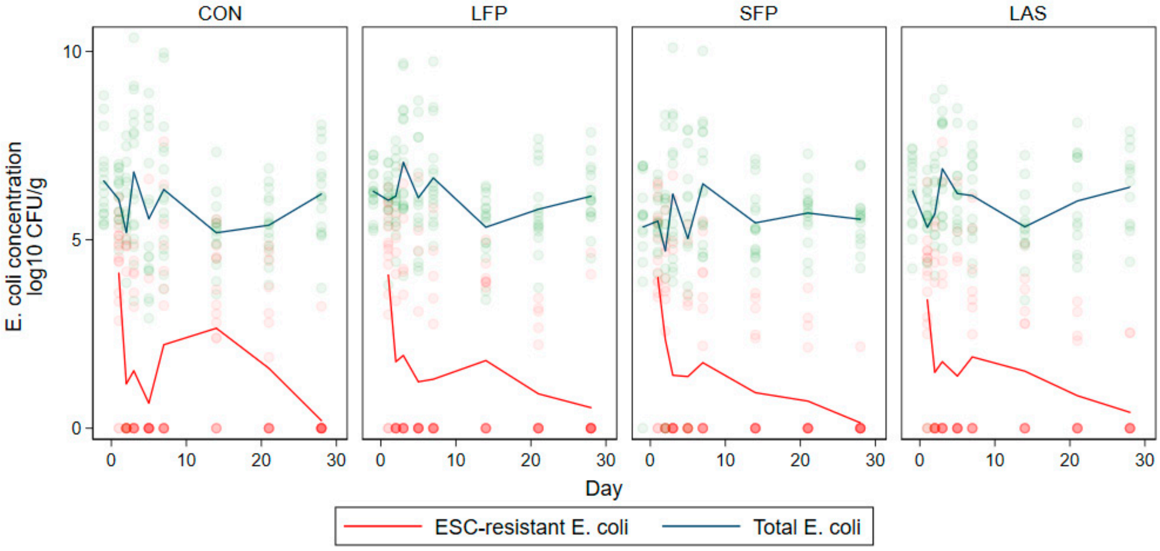

Supplementary Figure S1. Mean concentration of ESC-resistant *E. coli* and total *E. coli* in rectal swabs from ESC-resistant *E. coli* challenged weaners belonging to different treatment groups. Line represents median with individual pigs represented by dots (overlapping of individual pig data results in darker dots in graph). Treatment abbreviations: CON = control diet, LFP = CON + 2,000 ppm *Lactobacillus acidophilus* fermentation product (LFP), SFP = CON + 2,000 ppm *Saccharomyces cerevisiae* fermentation product (SFP), LAS = CON + 2,000 ppm LFP + 2,000 ppm SFP.

Supplementary Table S1. Calculated composition of experimental diets fed to ESC-resistant *E. coli* challenged weaners.

	CON	LFP	SFP	LAS
Ash (g/100g)	5.3	5.5	5.2	4.9
Crude Fibre (g/100g)	2.9	2.2	2.3	2.4
Total fat (g/100g)	4.5	2.4	4.9	4.8
Moisture (g/100g)	9.5	10.1	9.4	10.3
NFE (g/100g)	57.0	57.3	58.2	57.5

Treatment abbreviations: CON = control diet, LFP = CON + 2,000 ppm *Lactobacillus acidophilus* fermentation product (LFP), SFP = CON + 2,000 ppm *Saccharomyces cerevisiae* fermentation product (SFP), LAS = CON + 2,000 ppm LFP + 2,000 ppm SFP, NFE = nitrogen-free extract.
